# Beneficial Effect of Polysaccharide Gel Made of Xanthan Gum and Locust Bean Gum on Bovine Oocytes

**DOI:** 10.3390/ijms24043508

**Published:** 2023-02-09

**Authors:** Shunsuke Hara, Yuki Inoue, Sogo Aoki, Keisuke Tanaka, Koumei Shirasuna, Hisataka Iwata

**Affiliations:** Department of Animal Science, Tokyo University of Agriculture, Setagaya City 156-8502, Japan

**Keywords:** polysaccharide gel, oocyte maturation, TGFB1, F-actine, embryo development

## Abstract

The present study examined the effect of polysaccharides gels made of xanthan gum and locust bean gum (gel culture system) on oocyte maturation and explored the molecules causing the beneficial effect of the gel culture system. Oocytes and cumulus cells complexes were collected from slaughterhouse-derived ovaries and cultured on a plastic plate or gel. The gel culture system improved the rate of development to the blastocyst stage. The oocytes that matured on the gel contained high lipid contents and F-actin formation, and the resultant 8-cell stage embryos had low DNA methylation levels compared to their plate counterparts. RNA sequencing of the oocytes and embryos revealed the differentially expressed genes between the gel and plate culture systems, and upstream regulator analysis revealed estradiol and TGFB1 as top activated upstream molecules. The medium of the gel culture system contained higher concentrations of estradiol and TGFB1 than that of the plate cultures system. Supplementation of the maturation medium with either estradiol or TGFB1 resulted in high lipid content in oocytes. In addition, TGFB1 improved the developmental ability of the oocytes and increased F-actin content while reducing DNA methylation levels in the 8-cell stage embryos. In conclusion, the gel culture system is useful for embryo production, potentially through the upregulation of TGFB1.

## 1. Introduction

Oocyte maturation is an important step in the acquisition of developmental abilities. In vivo oocyte maturation occurs in follicular fluid, whereas in vitro, the condition for oocyte maturation is that of rigid plastic plates. In line with this difference, the quality of in vivo-matured bovine oocytes is better than that of in vitro-matured oocytes [1,2,3]. Follicular fluid contains proteoglycan and glycosaminoglycan at high concentrations, which have abundant hydroxyl bound with a negative charge. These molecules contribute to the osmotic pressure of follicular fluid [4] and works as a soft substrate; however, these properties have not been examined for oocyte maturation.

We developed a soft gel culture substrate made of polysaccharides, xanthan gum (XG), and locust bean gum (LBG), and reported that the gel culture system improved the in vitro growth of oocytes derived from immature small follicles (early antral follicles) and the in vitro development of early developmental stage bovine embryos [5,6]. Herein, the gel culture system activated genes associated with the focal adhesion and actine-mediated signal transduction; however, the key factors causing these changes are yet to be defined. RNA-sequencing (RNA-seq) technology combined with ingenuity upstream regulator analysis (IPA) is useful for identifying the molecules that cause the differential expression of genes in granulosa cells, embryos, and uterine epithelial cells [7,8,9].

In general, oocyte competence has been evaluated based on the health of the resultant offspring. However, this procedure is time-consuming and costly; therefore, oocyte quality is evaluated by maturation, fertilization, and developmental rate to the blastocyst stage, although these factors are not sufficient for enough to cover complete oocyte evaluation. Therefore, other factors, such as mitochondrial content, lipid content, and cytoskeletal distribution, have been used for oocyte evaluation [10,11,12,13]. In cows, total DNA methylation levels decreased until the 8-cell stage [14], and this demethylation is important for embryo development [15].

Here, we used the XG-LGB gels to improve oocyte maturation and examined its effect on oocyte quality using several developmental competence markers. In addition, we explored the upstream regulatory molecules responsible for the beneficial effect of the gel culture system using RNA-seq combined with IPA and examined the effect of these upstream molecules on the levels of developmental competence markers of oocytes.

## 2. Results

### 2.1. Gel Culture System Improves Oocyte Developmental Ability

Compared to plastic plates, culturing oocytes on a 1%-gel culture system significantly improved the rate of normal fertilization, and the rate of development to the blastocysts stage with a greater number of blastomeres. (Table 1). Based on this finding, the effect of the 1% gel culture system on oocytes was examined in subsequent experiments.

### 2.2. The Gel Culture System Increased Lipid Content and F-Actine Level and Lowered the Expression Levels of DNA Methylation in 8-Cell Stage Embryos

**Figure 1:**
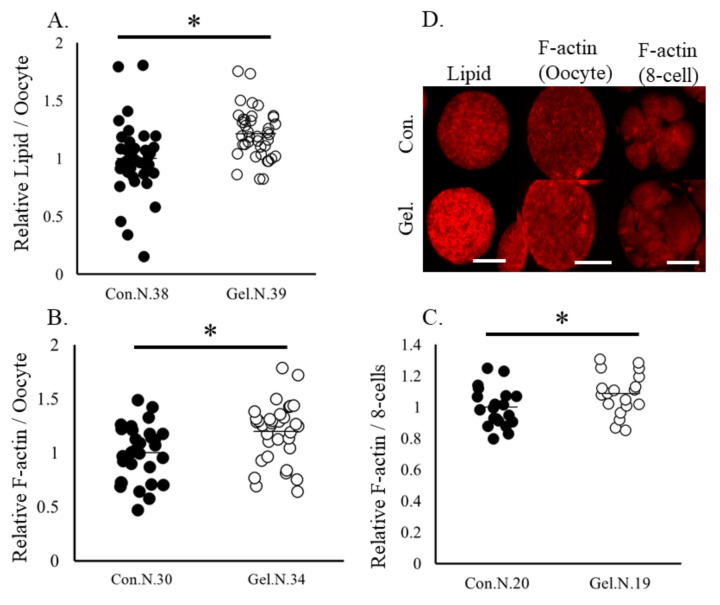
Lipid contents and levels of F-actin levels in oocytes and the 8-cell stage embryos. Oocytes were cultured on a plate (control) or a gel culture system (gel). (**A**), Lipid contents in oocytes. (**B**,**C**), F-actin levels in oocytes and 8-cell stage embryos. (**D**), Representative images of oocytes and 8-cell stage embryos. Data are normalized to that of the control. * *p* < 0.05. N, number of examined. Bar, 100 μm.

**Figure 2:**
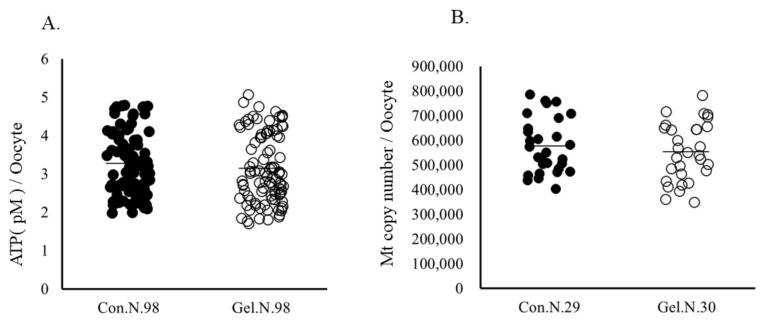
ATP (**A**) and mitochondrial DNA copy number (**B**) in oocytes. Oocytes were cultured on a plate (Control) or gel culture system (Gel). N, number of oocytes examined.

**Figure 3:**
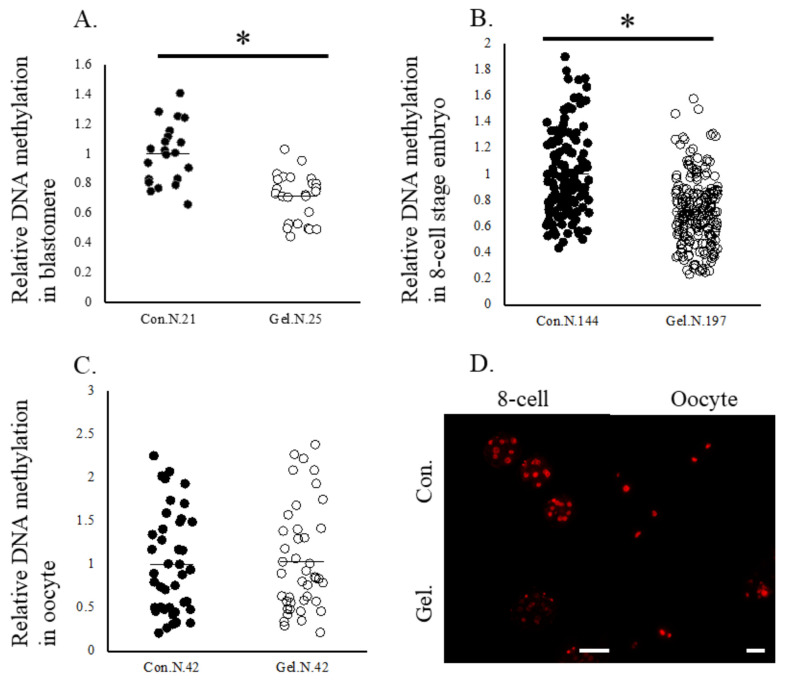
DNA methylation levels in oocytes and 8-cell stage embryos. Oocytes were cultured on plates (Control) or gel culture system (Gel) and matured oocytes and resultant 8-cell stage embryos were subjected to immunostaining. Relative expression levels of DNA methylation in blastomeres (**A**), in 8-cell stage embryos (**B**), and oocytes (**C**). Data are normalized to that of the control. * *p* < 0.05. N, number of oocytes examined. (**D**), representative pictures of oocytes and 8-cell stage embryos. Bar, 100 μm.

Oocytes matured on 1%-gel had significantly higher lipid contents and F-actine abundance, as well as higher F-actin abundance at the 8-cell stage compared with those cultured on plastic plates (Figure 1). Mitochondrial DNA copy number and ATP content in oocytes did not differ between the two culture systems (Figure 2). Furthermore, the immunostaining of 5-methyl cytosine revealed that the levels of DNA methylation were lower in whole embryos and blastomeres derived from gel culture systems than in those derived from the plate culture system, whereas DNA methylation levels in oocytes did not differ between the two age groups (Figure 3).

### 2.3. RNA-Seq of Oocytes, Cumulus Cells and 8-Cell Stage Embryos

RNA-seq revealed total of 1277 differentially expressed genes (DEGs) in cumulus cells cultured on plates and gels. A pathway analysis showed 35 significantly enriched pathways (all pathways are shown in Table S1). The top 10 pathways, including ECM-receptor interaction, MAPK signaling pathway, focal adhesion, and steroid biogenesis, among others, are presented in Table 2. Among the genes associated with ECM-receptor interaction, ITGAV and ITGB3 were upregulated in both oocytes and cumulus cells in the gel culture system (Table 3). Identification of upstream molecules that have the same directional influence on the DEGs in cumulus cells in the gel culture system using IPA analysis revealed that beta-estradiol and TGFB1 were the most top activated upstream regulators (Table 4). All pathways significantly enriched by the DEGs are shown in Table S2.

A pathway analysis revealed that ribosome, Parkinson’s disease, Huntington’s disease, oxidative phosphorylation, etc., were the significantly enriched pathways associated with the 1727 DEGs in oocytes matured on plates and gels (Top 10 in Table 2, all pathways associated with the DEGs are shown in Table S3). An IPA analysis showed that MYC, beta-estradiol, and TGFB1 are the activated upstream regulators of the DEGs in oocytes cultured on the gel culture system (The top 20 are shown in Table 5 and all significant upstream regulators are shown in Table S4).

An RNA-seq of 8-cell stage embryos revealed 791 DEGs; the significantly enriched pathways associated with these DEGs are shown in Table 6, including cell adhesion molecules, ECM-receptor interaction, PI3K-Akt signaling pathway, and mTOR signaling pathway. The expression levels of DNMT3A in embryos derived from the gel culture system were significantly lower (−1.7 fold, *p* < 0.05), and those of DNMT1 were lower (−1.3 fold and *p* = 0.055) than those in the plate counterparts. In contrast, the expression values of TET1/3 did not differ between the two groups (Table 4).

### 2.4. Effect of the Estradiol and TGFB1 on In Vitro Maturation (IVM) Medium on Oocytes

**Figure 4:**
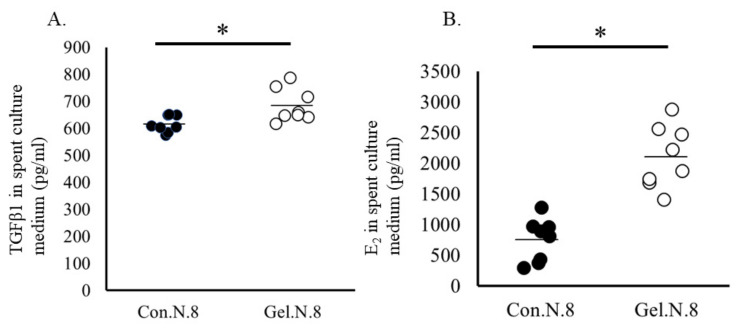
Concentration of TGFB1 (**A**) and estradiol (**B**) in the spent culture medium. Oocytes were cultured on a plate (Control) or gel culture system (Gel) * *p* < 0.05. N, number of oocytes examined.

**Figure 5:**
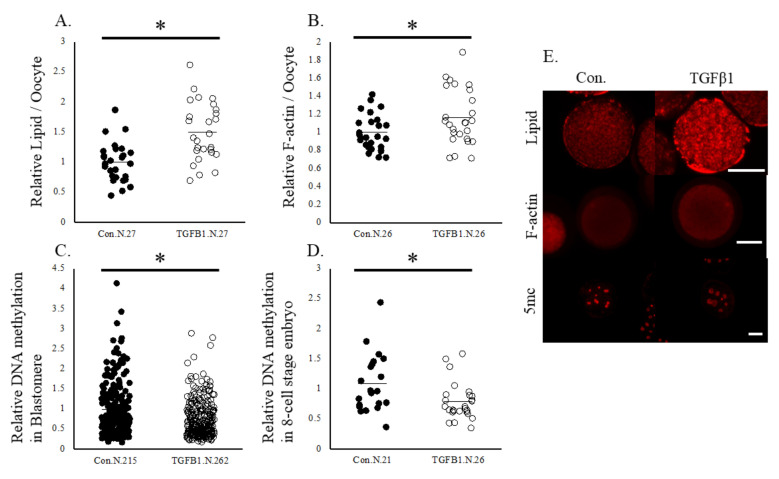
Lipid contents, levels of F-actin, and DNA methylation in oocytes and the 8-cell stage embryos. Oocytes were cultured in a medium containing vehicle (Control) or TGFB1 (50 ng/mL). (**A**), Lipid contents in oocytes. (**B**), levels of F-actin in oocytes. (**C**,**D**), levels of DNA methylation in blastomere and the 8-cell stage embryos. (**E**), representative pictures of oocytes and 8-cell stage embryos. Data are normalized to that of the control. * *p* < 0.05. N, number of oocytes examined. Bar, 100 μm.

**Figure 6:**
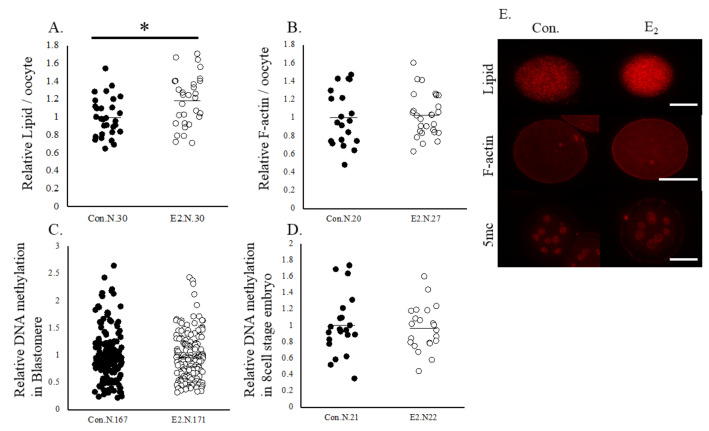
Lipid contents, levels of F-actin, and DNA methylation in oocytes and the 8-cell stage embryos. Oocytes were cultured in a medium containing vehicle (Control) or estradiol(100 ng/mL). (**A**) Lipid contents in oocytes. (**B**) Levels of F-actin in oocytes. (**C**,**D**) Levels of DNA methylation in blastomere and the 8-cell stage embryos. (**E**) Representative pictures of oocytes and 8-cell stage embryos. Data are normalized to that of the control. * *p* < 0.05. N, number of oocytes examined. Bar, 100 μm.

Based on the IPA of the DEGs in oocytes and cumulus cells, we selected overlapped upstream regulators, beta-estradiol and TGFB1, and examined whether beta-estradiol and TGFB1 affect oocytes in the same way that is observed in oocytes cultured in a gel culture system. First, the spent IVM medium in the gel culture system contained higher concentrations of estradiol and TGFB1 than that in the plate culture system (Figure 4). Supplementation with 50 ng/mL TGFB1 significantly increased the rate of development to the blastocyst stages (Table 7). Additionally, TGFB1 significantly increased the expression levels of F-actin and lipid content in oocytes. In addition, TGFB1 reduced the levels of 5 mc in the 8-cell stage embryos and their blastomere (Figure 5). Supplementation of IVM medium with 100 ng/mL estradiol increased lipid content in oocytes but did not affect the developmental rate of the oocytes (Table 7), F-actin content in oocytes, or levels of methylation in 8-cell stage embryos (Figure 6).

## 3. Discussion

The present study showed that the XG-LBG gel culture system used for IVM improved the ability of oocytes to develop to the blastocyst stage. In addition, the oocytes showed upregulation of F-actin formation, accumulation of lipids in oocytes, and low DNA methylation levels in 8-cell stage embryos. An RNA-seq of oocytes and cumulus cells showed that beta-estradiol and TGFB1 are potential mediators of the effect of the gel culture system. Supplementation of culture medium with TGFB1 increased F-actin expression and lipid content and decreased methylation levels in 8-cell stage embryos.

The XG-LGB gel had abundant hydroxyl oxide-bound residues and was negatively charged, with soft and low adhesive character to the cellular membrane; consecutively, granulosa cells cultured on the gel did not attach to the gel substrate and formed a spheroid cell mass [6]. In general, cells proliferate on a stiff substrate through focal adhesion activation and ECM–cell interactions, and this conventional notion cannot explain our findings that cumulus cells and oocytes cultured on soft and less adhesive XG-LGB gel promoted focal adhesion and ECM receptor interactions in both cells and oocytes. Mandal et al. [16] reported that cells cultured on soft hyaluronic gel upregulated PI3K signaling and actin formation, which increased membrane traction force and cell membrane tension, similar to that seen on a stiff culture substrate such as polyacrylamide. In line with this, PI3K signaling was a top enriched pathway associated with the DEGs in cumulus cells; however, whether the same mechanism works in gel–cell interaction remains unclear. Our previous report showed that embryos cultured on the XG-LGB gel upregulated actin formation and cadherin localization [5], and oocyte granulosa cell complexes derived from early antral follicles cultured on the XG-LGB gel upregulated genes associated with focal adhesion and ECM interactions [6]. Gel substrate then transfers certain signals to activate focal adhesion and ECM interaction-related molecules on cumulus cells and oocytes.

Integrin adhesion complexes are located in the extracellular matrix, plasma membrane, and cytoskeleton, and play an important role in mechano-transduction, where cells sense forces derived from ECM and cellular contraction and convert them into biochemical signals [17]. A RNA-seq analysis showed that the expression levels of *ITGAV* and *ITGB3* were significantly upregulated in both oocytes and granulosa cells on the gel. In addition, luminal pressure induces cortical tension and increases cell–cell adhesion and tight junction, which change embryonic fate [18]. Therefore, integrin and mechanical pressure may mediate interactions between gel substrate and cumulus cell-oocyte complexes (COCs); however, it is difficult to deduce the molecules using limited information. To explore the molecules mediating the interaction among gel substrate, oocytes, and cumulus cells, we conducted an IPA analysis to predict upstream regulators causing change in gene expression in the same direction as the DEGs using the vast accumulating information. TGFB1 and estradiol were predicted as activated molecules of the DEGs in both oocytes and cumulus cells cultured on gel. Consistent with this prediction, we found that the two molecules were present at a significantly higher concentration in the spent culture medium of the gel culture system than in their plate counterparts. Steroid biosynthesis was the most enriched pathway of the DEGs, where genes associated with cholesterol and steroid biogenesis were significantly upregulated in cumulus cells cultured on gel. Su et al. [19] showed that GDF9 derived from oocyte induced cholesterol biosynthesis in cumulus cells. Consistent with this report, the present RNA-seq showed significantly high GDF9 expression in oocytes cultured on gel (1.2 fold, *p* < 0.006).

High TGFB1 in large antral follicles compared with those in small follicles and localization between the zona pellucida and ooplasm have been reported in pigs [20]. In our RNA-seq data, the expression levels of TGFB1 mRNA did not differ between gel and plate systems (oocyte: *p* = 0.87, cumulus cells: *p* = 0.68), indicating the presence of post transcriptional modification or other mechanisms regulating free TGFB1 in the culture medium. In line with this, it has been reported that latent TGFB1 is stored in TGFB-binding proteins (LTBP), and cellular contracting force liberates TGFB1, which binds to the TGFB1 receptor [21].

Lipid content in bovine oocytes is associated with high developmental competence [11]. Gel, TGFB1, and estradiol significantly increased the lipid content in oocytes, suggesting that TGFB1 and estradiol are potential mediators of the effect of the gel culture system on oocyte lipid content. Based on the RNA-seq data of the oocytes, gel induced low lipid metabolism (BECR CPT1A) and high fatty acid elongation (SCD and FADS2, Table 3); whether a similar mechanism works under supplementation with estradiol or TGFB1 needs further validation.

The mitochondrial number and functions are crucial markers of oocyte competence [22,23]. Although oxidative phosphorylation is the most enriched pathway in oocytes cultured on gel, differential mitochondrial number and function were not detected in oocytes between gel and plate culture systems, and require further evaluation.

To date, there have been no reports regarding the relationship between the amount of F-actin in oocytes and developmental competence, although embryos developed on XG-LBG gel showed increased levels of F-actin [5]. In the present study, either the gel culture system or TGFB1 increased F-actin formation in oocytes and improved developmental competence. In line with this, the upregulation of F-actin formation by TGFB1 treatment has been reported in MSC, peritoneal fibroblasts, and SiHa cells [24,25,26]. Supporting this, supplementation of the IVM medium with specific inhibitors of TGFB1 (SD208) significantly decreased the F-actin content in mature oocytes (Supplementary Figure S1).

Another question raised in the present study was the quality of embryos derived from oocytes cultured on the gel. Owing to improve in vitro embryo production methods, embryos produced in vitro has been widely used for calf production. However, high methylation of in vitro-produced 8-cell stage embryos [27] and abnormal DNA methylation in blastocysts induced by in vitro embryo culture have been reported [28]. In cows, it has been reported that methylation levels of embryo decrease during the 2–8 cell stage and remain low until the blastocyst stage [14]. Surprisingly, at the 8-cell stage, when zygotic genome activation occurs [29], embryos derived from oocytes matured on gel had low DNA methylation levels and these results were reproduced by supplementation of IVM medium with TGFB1. This study is the first to report that oocyte maturation conditions reduce DNA methylation status during zygotic genome activation (around the 8-cell stage in cows). We further explored gene expression in 8-cell stage embryos derived from the gel culture system. An RNA-seq showed that compared to those cultured using plate culture system, *DNMT3A* was expressed at significantly lower levels and *DNMT1* tended to be expressed at lower levels in embryos derived from the gel culture system, which is a possible background for low DNA methylation. Furthermore, the DEGs were associated with ECM receptor interaction and cell adhesion molecules, indicating that the influence of gel culture system remains in early cleaved-stage embryos. A complete analysis of the resultant blastocysts requires further experimentation. Nevertheless, two embryo transfers were conducted and two healthy cows were obtained, and they grew normally (Figure S2).

## 4. Materials and Methods

### 4.1. Ovary Collection

Bovine ovaries were collected at a local slaughterhouse and transported to the laboratory in phosphate-buffered saline containing antibiotics at 25 °C within 4 h. Cumulus cell-oocyte complexes (COCs) were aspirated from the antral follicles (3–5 mm in diameter) using an 18 G needle (Terumo, Tokyo, Japan) connected to a 10 mL syringe (Terumo). Bovine ovaries were routinely discarded at the slaughterhouse; therefore, they were donated to us for this study. The study was approved by the Ethics Committee for Animal Experiments of the Tokyo University of Agriculture.

### 4.2. Chemicals and Media

All chemicals were purchased from Nacalai Tesque (Kyoto, Japan), unless otherwise described. Medium 199 (Gibco, Grand Island, NY, USA) supplemented with 10 mM taurine, 10 ng/mL EGF (Sigma, St. Louis, MO, USA), 100 IU/mL penicillin, 0.1 μg/mL streptomycin, and 10% fetal calf serum (FCS; 5703H; ICN Pharmaceuticals, Costa, Mesa, CA, USA) was used for IVM of oocytes. Media for IVF and IVC were synthetic oviductal fluid (SOF) with some modifications as described in [7]. The IVF medium consisted of SOF supplemented with 4 mg/mL fatty acid-free bovine serum albumin (BSA) and 10 IU/mL heparin. The medium used for the IVC of embryos comprised SOF supplemented with essential and non-essential amino acids (Sigma-Aldrich, St. Louis, MO, USA), 5% FCS, and 1.5 mM glucose. At 48 h post-fertilization, cleaved embryos were denuded from surrounding cumulus cells and the embryos were transported to a new drop (10–15/drop) and subsequently cultured for 5 days. IVM and IVF, and IVC for 48 h post insemination were conducted under conditions of 5% CO_2_ and 95% air at 38.5 °C. Two days after insemination, the embryos were cultured under the atmospheric conditions of 5% CO_2_, 5% O_2_, and 90% air at 38.5 °C.

### 4.3. Preparation of Gel and IVM

**Figure 7:**
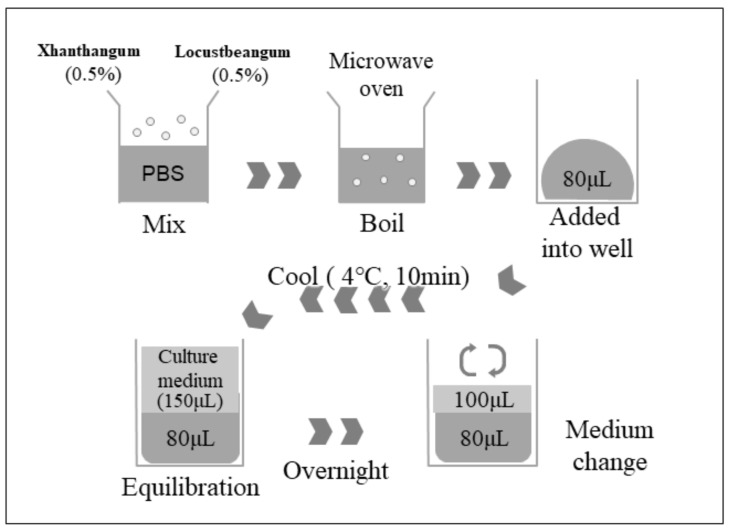
In belief, each 0.5% of XG and LBG were mixed in PBS and heated in a microwave oven until boiling. Complete melted XG and LBG (80 μL) was added into each well of a 96-well plate (Falcon) and then cooled to form gels. The gel was equilibrated with IVM medium overnight, and the medium was changed with fresh IVM medium before experiment.

Polysaccharide gel consisting of xanthan gum (XG) and locust bean gum (LBG) (Sansho Co., Ltd., Osaka, Japan) was prepared as reported previously [5] (see in detail in Figure 7). For the IVM of oocytes, 100 μL of IVM medium was placed on gel substrate or on plastic plate without gel (10 COCs/well). Concentration of the gel was determined based on the previous reports [5,6].

### 4.4. IVF and IVC of Embryos

COCs were cultured for 21 h on gel or plastic plates, and then fertilized. Frozen thawed semen from a bull was washed with the discontinuous gradient Percoll solution (Cytiva Tokyo, Japan, 30, and 60% in IVF medium) and co-incubated with COCs for 6 h. Oocytes were then transferred to the IVC medium and cultured for 40 h with surrounding cumulus cells. Then, cleaved embryos were denuded from the cumulus cells and cultured in IVC droplets (50 μL) for 5 days. Seven days post-insemination, the blasturation rate was recorded, and total cell number of the blastocysts was examined.

Fertilization was determined by treatment with a presumptive zygote with actetic ethanol (1:3) for 5 min. Zygotes with two pronucleus were considered to have undergone normal fertilization. The total cell number of the blastocysts was determined by Hoechst 33342 staining under a fluorescence microscope (Keyence, Tokyo, Japan).

### 4.5. Measurement of Lipid Content in Oocytes

After IVM for 21 h, oocytes were denuded and fixed in 4% paraformaldehyde in PBS overnight, and lipid content was determined by Nile red staining (Wako, Tokyo, Japan) for 10 min. Oocytes were mounted onto glass slides using an anti-fade reagent containing DAPI (Invitrogen, Waltham, MA, USA). Oocytes were observed under a Leica DMI 6000B microscope using LAS AF software (Leica, Wetzlar, Germany), and fluorescence intensities of the equatorial region of oocytes were quantified using ImageJ software (NIH, Bethesda, MD, USA).

### 4.6. Evaluation of F-Actin

After IVM for 21 h, the oocytes were denuded and fixed in 4% paraformaldehyde in PBS overnight, followed by permeabilization (0.25% TritonX) and blocking in 4% BSA/PBS. F-actin was stained using Actin-stain 555 phalloidin (Cytoskeleton, Denver, CO, USA) for 30 min. Oocytes were mounted and observed as described above.

### 4.7. Immunostaining

Oocytes after IVM for 21 h and cleaved embryos (48 h post insemination) were randomly collected and subjected to immunostaining. The cells were fixed in 4% paraformaldehyde in PBS overnight, and then permeabilized in (0.25% TritonX) for 30 min. Embryos were treated with 2N HCl for 1 h at 37 °C, followed by blocking with 4% BSA/PBS. The primary antibody used was rabbit monoclonal antibody (D3S2Z, Cell Signaling, Danvers, MA, USA, ×200 in Blocking solution) and the secondary antibody used was anti-Rabit IgG Fab2 Alexa Flor 555 (Cell Signaling). Embryos were observed under a Leica DMI 6000B microscope using LAS AF software (Leica, Wetzlar, Germany), and the fluorescence intensities of the nucleus were quantified using ImageJ software (NIH, Bethesda, MD, USA). The fluorescence intensity of each blastomere and or spindle (oocyte) was then measured. In addition, the average value for each embryo was calculated.

### 4.8. Measurement of mtDNA Number and ATP Content in Oocytes

The ATP content in individual oocytes were measured using an ATP assay kit (Toyo-Inc., Tokyo, Japan), as previously described [30]. Luminescence generated with ATP-dependent luciferin-luciferase was measured using a luminometer (Spark 10 M; Tecan Japan Co., Ltd., Kanagawa, Japan). The mitochondrial DNA copy number in oocytes were measured by quantitative PCR using serial dilution of standard as previously described [30]. DNA of each oocyte was extracted by heating at 55 °C for 30 min and at 98 °C for 10 min in extraction buffer (Tris-HCl, 20 mM containing Nonidet P-40, 0.9% Tween 20, and 0.9% proteinase K, 0.4 mg/mL). PCR was conducted using the CFX ConnectTM Real Time PCR system (Bio-Rad, Hercules, CA, USA), KAPA SYBER FAST qPCR Kit (Roche, Indianapolis, IN, USA) and specific primers. The primer sets used to determine mtDNA number were 5-ACCCTTGTACCTTTGCAT-3 and 5-TCTGGTTTCGGGCTCGTTAG-3 (81 bp, NC_006853.1). The PCR program was 95 °C for 3 min, followed by 40 cycles of 98 °C for 5 s and 60 °C for 10 s. The quality of the PCR products was checked using melt analysis, and the size of the PCR products was determined by electrophoresis. A standard was PCR products of the corresponding gene cloned into a vector. The vector containing corresponding the PCR product was sequenced before use, and the DNA copy number was calculated using Avogadro’s constant and the molar quantity of the standard vector. The PCR efficiency was >1.99.

### 4.9. Measurement of Estradiol and TGFB1 Concentration in Spent Culture Medium

The spent culture medium was collected after IVM. The gel could contain estradiol and TGFB1; therefore, the total sample volume was adjusted to 180 μL (100 μL of IVM spent culture medium plus 80 μL of melted gel). For the gel culture system, the corresponding spent gel, for the plate system, gel incubated in the culture medium without COCs. This gel was transferred into PCR tube followed by incubation at 80 °C for 5 min. The melted gel and the spent culture medium were mixed and cooled to room temperature. These samples were subjected to the measurement using an Estradiol Assay kit (R&D Systems, Minneapolis, MN, USA) and TGFB1 ELISA Kit (Legend Max TM, Biolegend San Diego, CA, USA).

### 4.10. RNA-Seq

Seventy COCs were cultured on plates or gel for 21 h and then the oocytes and cumulus cells were collected for RNA extraction. In addition, forty 8-cell stage embryos were produced using a plate or gel culture system. Three samples were prepared for each experimental group using a differential ovary series. RNA extraction was conducted using RNAqueous™-Micro (Thermo Fisher, Waltham, MA, USA), and the quality and concentration of the total RNA were determined using a 2100 Bioanalyzer (Agilent Technologies, Santa Clara, CA, USA). The RNA quality index was 7.7 ± 1.1. cDNA libraries of the RNA from oocytes and cumulus cells were prepared using the SMART Seq v4 Ultra^®^ Low Input RNA Kit for Sequencing (Clontech, Mountain View, CA, USA). cDNA libraries of the RNA of cumulus cells were prepared using the NEBNext Ultra II RNA Library Prep Kit for Illumina (New England Biolabs, Ipswich, MA, USA). The concentration of the cDNA libraries was determined using an Agilent high sensitivity DNA kit and a Bioanalyzer 2100 (Agilent Technology, Santa Clara, CA, USA). The concentration of the cDNA libraries was reassessed using the Kapa Library Quantification kit (Kapa Biosystems, Wilmington, MA, USA). The multiplexed sample was sequenced in single-read 75 bp reads on the NextSeq 500 system (Illumina).

For the RNA-seq of 8-cell stage embryos, cDNA libraries of the RNA were prepared using theNEBNext Single Cell/Low Input RNA Library Prep Kit for Illumina (New England Biolabs). The concentration of the cDNA libraries was determined using an Agilent high sensitivity DNA kit and a Bioanalyzer 2100 (Agilent Technology). The concentration of the cDNA libraries was reassessed using the Kapa Library Quantification kit (Kapa Biosystems). The multiplexed sample was sequenced in single-read 100 bp reads on the NextSeq1000 system (Illumina).

Raw data were generated using the bcl2fastq2 software(Illumina), according to the manufacturer’s instructions. The process of sequence preparation, reference genome mapping, and differential gene expression analysis were performed using CLC Genomics Workbench ver. 22.0.2 (Qiagen, Hilden, Germany). To prepare the sequenced data, adapter sequences, ambiguous nucleotides, and low-quality sequences were removed from the data. The remaining sequence data were aligned to the Bos taurus genome sequence (ARS-UCD1.2/bosTau9) to count sequence reads. Gene expression values were evaluated using reads per kilobase of transcript per million mapped reads (RPKM). The differential expressed genes (DEGs) were determined using transcriptomic tools from the CLC Genomics Workbench with a threshold of *p* value < 0.05.

Pathways from the according to the Kyoto Encyclopedia of Genes and Genomes (KEGG) enriched by DEGs were predicted using a functional annotation tool (DAVID, https://david.ncifcrf.gov accessed on 7 February 2023), using *Bos taurus* as the background and species. The upstream regulator function of the Ingenuity Pathway Analysis software (IPA, Qiagen) was used to predict the upstream transcriptional regulators of DEGs. This method was used to determine the number of known targets of each transcriptional regulator present in the DEGs. Overlapping *p*-values were calculated to measure significant overlap. Raw data of RNA-seq data of oocytes, cumulus cells, and 8-cell stage embryos have been registered in DDBJ under the accession numbers DRA015240 and DRA015241.

### 4.11. Effect of Supplementation of the IVM Medium with Estradiol on Oocytes

Estradiol was diluted with ethanol (×2000) and added to the IVM medium (final concentrations of E2 were 50 ng/mL and 500 ng/mL). TGFB1 was diluted in water (×1000) and added to the IVM medium (final concentration of TGFB1 was 50 ng/mL. The concentration of TGFB1 was determined based on the developmental rate to the blastocyst stage (0, 10 and 50 ng/mL). The concentration of beta-estradiol was determined based on the lipid contents in in vitro matured oocytes (0, 10 and 100 ng/mL), where 100 ng estradiol significantly increased the lipid content. The IVM medium was modified, wherein FCS was replaced with 3 mg/mL BSA. Oocyte maturation, fertilization, and development was conducted as described above. The effects of estradiol or TGFB1 on lipid content, F-actin formation in oocytes, levels of 5 mC in the 8-cell stage embryos, and developmental rate to the blastocyst stage were examined as described above.

### 4.12. Statistical Analysis

All data are presented as the mean ± standard error of the mean (SEM). All data were analyzed using the Shapiro–Wilk test followed by Student’s *t*-test, and nonparametric data were analyzed using the Mann–Whitney U test. Percentages of fertilization and blastulation rates were arcsine-transformed prior to analysis. Statistical significance was set at *p* < 0.05.

## 5. Conclusions

Polysaccharide gel made an XG-LCB gel is a useful material for the IVM of bovine oocyte, promoting F-actin formation and lipid contents, as well as reducing DNA methylation in 8-cell-stage embryos. The beneficial effect of XG-LBG gel is likely associated with TGFB1 signaling.

## Figures and Tables

**Table 1:** Effect of gel culture system on oocytes maturation and subsequent embryo development.

Group	In Vitro Maturation			In Vitro Fertilization			In Vitro Culture					
	**No. of**		**Rate of**	**No. of**		**Rate of**	**No. of**		**Rate of**		**Blastocysts**	
	**Trials**	**Oocytes**	**M2 (%)**	**Trials**	**Oocytes**	**Normal (%)**	**Trials**	**Oocytes**	**Cleaved**	**Blastocyst**	**No.**	**TCN**
Con	7	70	72.9 ± 5.2	12	111	87.1 ± 3.0 a	9	90	55.6 ± 2.4	14.4 ± 2.4 a	14	113.1 ± 7.6 a
1%	7	70	82.9 ± 6.1	12	121	93.9 ± 1.7 b	9	100	55.6 ± 3.1	25.7 ± 4.1 b	26	138.3 ± 8.0 b
2%	7	67	65.9 ± 6.1	12	117	86.8 ± 3.1 ab	9	94	44.4 ± 2.4	14.1 ± 3.4 a	10	81.8 ± 7.2 c

Data are represented as means ± SEM. After in vitro maturation (IVM) on gel (1% or 2%) or plate (Con), the rate of oocytes at metaphase 2 (M2), normal fertilization rate (Normal), and rate of embryos developed to the blastocyst stage and total cell number (TCN) of the blastocytes 7 days after insemination was examined. different script, a–c, *p* < 0.05.

**Table 2:** Pathway associated with differentially expressed genes in cumulus cells and oocytes between the plate and gel culture systems.

Term	Count	*p*-Value
Cumulus cells		
ECM-receptor interaction	24	4.49 × 10^−9^
MAPK signaling pathway	44	1.74 × 10^−7^
Pathways in cancer	67	1.85 × 10^−7^
Focal adhesion	34	4.03 × 10^−7^
Steroid biosynthesis	10	1.45 × 10^−6^
Arrhythmogenic right ventricular cardiomyopathy	18	4.16 × 10^−6^
AGE-RAGE signaling pathway in diabetic complications	20	2.25 × 10^−5^
Rap signaling pathway	30	0.000112
Human papillomavirus infection	42	0.000116
Metabolic pathways	137	0.00015
Oocyte		
Ribosome	47	3.72 × 10^−14^
Parkinson disease	57	1.79 × 10^−10^
Huntington disease	60	1.06 × 10^−9^
Pathways of neurodegeneration—multiple diseases	80	1.38 × 10^−9^
Coronavirus disease—COVID-19	54	3.84 × 10^−9^
Prion disease	51	8.60 × 10^−8^
Amyotrophic lateral sclerosis	62	1.85 × 10^−7^
Alzheimer disease	62	1.03 × 10^−6^
Thermogenesis	43	1.36 × 10^−6^
Oxidative phosphorylation	30	3.09 × 10^−6^
Chemical carcinogenesis—reactive oxygen species	40	1.98 × 10^−5^

**Table 3:** Comparison of the expression levels of genes of interest in 8-cell stage embryos, in vitro matured oocytes, and the corresponding cumulus cells.

Name	Max Group Mean	Fold Change	*p*-Value
	RPKM	Gel/plate	
8 cell-stage embryo			
DNMT3B	3.281	1.381	0.323
DNMT3A	2.189	−1.725	0.049
DNMT1	31.342	−1.330	0.056
TET1	0.128	−1.049	0.960
TET2	6.817	−1.271	0.180
TET3	3.000	−1.350	0.192
Oocytes			
CPT1A	10.793	−1.163	0.039
FADS2	0.316	2.800	0.010
GDF9	399.813	1.212	0.006
ITGAV	5.460	1.967	0.002
ITGB3	1.921	1.666	0.001
MECR	0.852	−1.617	0.027
SCD	9.917	2.379	0.004
TGFBI	2.130	−1.095	0.874
Cumulus cells			
CYP19A1	0.913	2.150	0.002
ITGB3	125.190	1.290	0.002
ITGAV	272.418	1.220	0.028
TGFB1	22.786	1.040	0.675

**Table 4:** Upstream regulators of DEGs in cumulus cells cultured on gel culture systems.

Upstream	Molecular	Predicted	Activation	*p* Value
Regulator	Type	Activation State	Z-Score	of Overlap
beta-estradiol	chemical-endogenous mammalian	Activated	5.826	3.17 × 10^−50^
TGFB1	growth factor	Activated	7.165	2.93 × 10^−47^
dexamethasone	chemical drug	Activated	4.082	1.61 × 10^−42^
TNF	cytokine	Activated	4.627	4.53 × 10^−42^
AGT	growth factor	Activated	6.23	2.05 × 10^−34^
CG	complex	Activated	4.499	1.91 × 10^−33^
PD98059	Chemical: kinase inhibitor	Inhibited	−5.238	9.41 × 10^−32^
lipopolysaccharide	chemical drug	Activated	6.403	2.66 × 10^−31^
IFNG	cytokine	Activated	5.852	1.02 × 10^−30^
tretinoin	Chemical: endogenous mammalian	Activated	3.597	3.6 × 10^−28^
IL1B	cytokine	Activated	5.168	6.91 × 10^−28^
LY294002	Chemical: kinase inhibitor	Inhibited	−5.177	2.22 × 10^−27^
progesterone	Chemical: endogenous mammalian	Activated	2.93	4.47 × 10^−27^
ESR2	ligand-dependent nuclear receptor		1.965	5.74 × 10^−27^
U0126	Chemical: kinase inhibitor	Inhibited	−4.479	3.32 × 10^−25^
STAT3	transcription regulator	Activated	2.553	6.35 × 10^−25^
PDGF BB	complex	Activated	4.112	1.66 × 10^−24^
MAP2K5	kinase	Activated	4.991	2.57 × 10^−24^
TGFB	group	Activated	3.897	3.03 × 10^−24^
TP53	transcription regulator		1.597	3.79 × 10^−24^

DRGs: differentially expressed genes. Activated: activated upstream regulator causing the differential expression of genes in the same direction. Inhibited: Inhibited upstream regulator causing the differentially expressed genes in the opposite direction.

**Table 5:** Upstream regulator of DEGs in oocytes cultured on gel culture systems.

Upstream	Molecular	Predicted	Activation	*p* Value
Regulator	Type	Activation State	Z-Score	of Overlap
MYC	transcription regulator	Activated	7.392	3.42 × 10^−21^
MYCN	transcription regulator	Activated	3	9.15 × 10^−21^
beta-estradiol	chemical-endogenous mammalian	Activated	3.75	1.14 × 10^−20^
torin1	chemical reagent	Inhibited	−4.543	1.32 × 10^−19^
Lh	complex	Activated	4.001	3.15 × 10^−19^
TP53	transcription regulator		1.571	3.73 × 10^−19^
LARP1	translation regulator	Inhibited	−5.743	2.63 × 10^−18^
APP	other		0.412	3.84 × 10^−16^
CTNNB1	transcription regulator		1.635	1.24 × 10^−15^
TGFB1	growth factor	Activated	2.748	1.61 × 10^−14^
MAPT	other		0	1.98 × 10^−14^
RICTOR	other	Inhibited	−6.308	5.72 × 10^−14^
ESR1	ligand-dependent nuclear receptor	Activated	3.228	5.94 × 10^−13^
ST1926	chemical drug	Inhibited	−5.492	1.22 × 10^−12^
tretinoin	chemical-endogenous mammalian		−0.169	2 × 10^−12^
acyline	biologic drug			4.67 × 10^−12^
ESR2	ligand-dependent nuclear receptor	Activated	2.958	5.68 × 10^−12^
MLXIPL	transcription regulator	Activated	5.444	5.69 × 10^−12^
dihydrotestosterone	chemical-endogenous mammalian	Activated	2.984	3.36 × 10^−11^
decitabine	chemical drug		1.223	4.75 × 10^−11^

DEGs: differentially expressed genes. Activated: activated upstream regulator causing the differential expression of genes in the same direction. Inhibited: Inhibited upstream regulator causing the differentially expressed genes in the opposite direction.

**Table 6:** Pathway enriched by DEGs in 8-cell stage embryos.

Term	Count	*p*-Value
Nicotine addiction	7	0.0028997
Neuroactive ligand-receptor interaction	26	0.0037227
Cell adhesion molecules	14	0.0090386
Human papillomavirus infection	22	0.0166348
GABAergic synapse	9	0.0168932
Cytokine-cytokine receptor interaction	21	0.0179321
PI3K-Akt signaling pathway	23	0.0190965
PD-L1 expression and PD-1 checkpoint pathway in cancer	9	0.0201767
Type I diabetes mellitus	7	0.0207458
Taste transduction	8	0.0301596
Herpes simplex virus 1 infection	23	0.04698

**Table 7:** Effect of TGFB1 or E2 on the developmental ability of oocytes.

Group	No. of	No. of	Rate of
	Trials	Oocytes	Blast.
Con.	9	89	20.1 ± 2.2 a
TGFB1	9	89	30.2 ± 1.4 b
Con	9	90	28.9 ± 3.3
Estradiol	9	90	23.3 ± 3.1

Oocytes were cultured in a medium containing 50 ng/mL TGFB1 or vehicle (Con, water) or 100 ng/mL estradiol or vehicle (Con. Ethanol). a,b *p* < 0.05.

## Data Availability

Data of the comprehensive gene expression was registered at DDJB as (DRA015240 and DRA015241). All data for figure and table are available on proper request from the corresponding author.

## Supplementary Materials

The following supporting information can be downloaded at: https://www.mdpi.com/article/10.3390/ijms24043508/s1.
