# Supplementary material for: Beneficial Effect of Polysaccharide Gel Made of Xanthan Gum and Locust Bean Gum on Bovine Oocytes
Source: Int J Mol Sci. 2023 Feb 9;24(4):3508. doi: 10.3390/ijms24043508 (PMC9963600; doi:10.3390/ijms24043508)
Supplement: Supplementary file 1 [file ijms-24-03508-s001.zip › Figure S1.pdf]

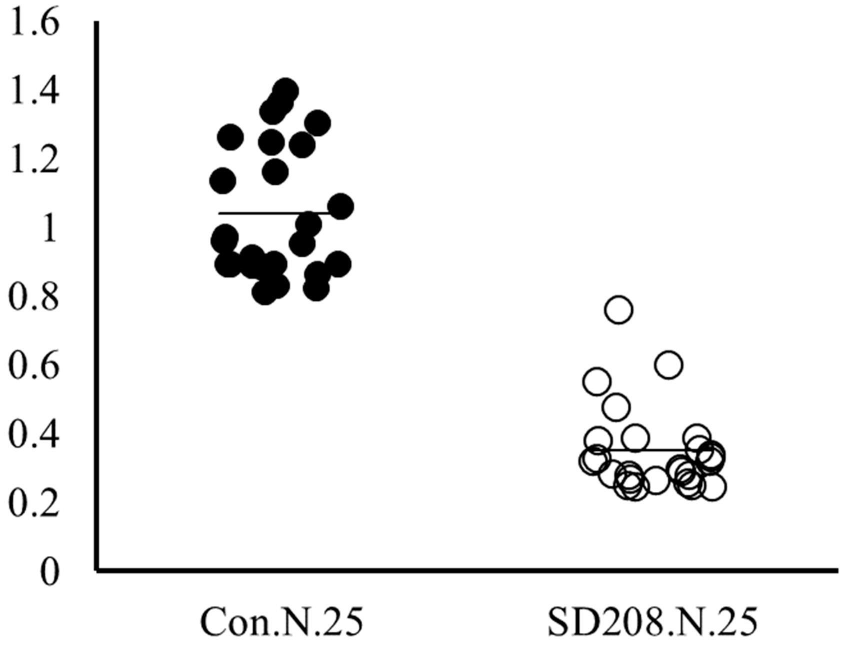

Figure S1. COCs were cultured in IVM medium with or without 2.5 μM of SD-208, a novel transforming growth factor beta receptor I kinase inhibitor for 21h and the denuded oocytes were examined F-actin formation as described above. Y axis: expression levels which was normalized by control. N. number of oocytes examined.
