# Supplementary figures and images for: Beneficial Effect of Polysaccharide Gel Made of Xanthan Gum and Locust Bean Gum on Bovine Oocytes
Source: Int J Mol Sci. 2023 Feb 9;24(4):3508. doi: 10.3390/ijms24043508 (PMC9963600; doi:10.3390/ijms24043508)

Figure S2

Cows derived from oocytes matured on the gel culture system.

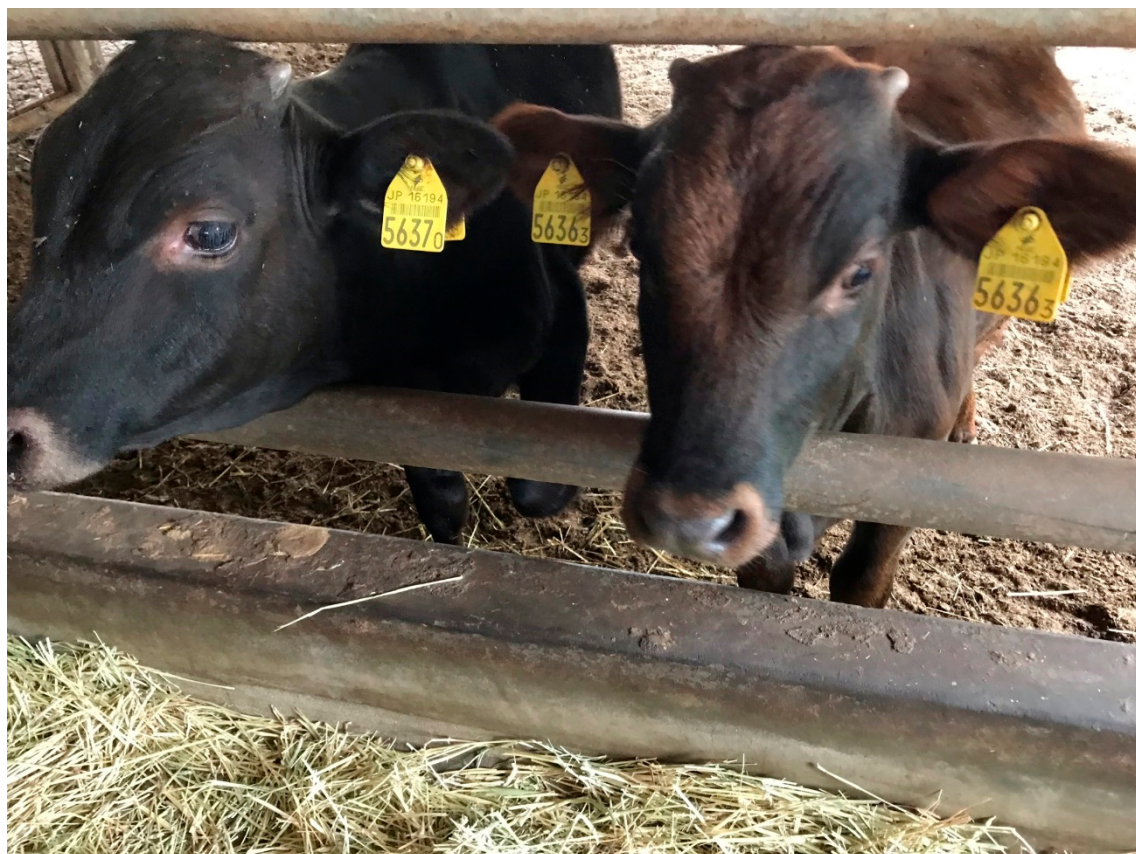

Supplement: Supplementary file 1 [file ijms-24-03508-s001.zip › Figure S2.pdf]
